# Evaluating Nuclear Factor NF-κB Activation following Bone Trauma: A Pilot Study in a Wistar Rats Model

**DOI:** 10.1371/journal.pone.0140630

**Published:** 2015-10-14

**Authors:** Marcos Barbosa Salles, Sergio Alexandre Gehrke, Jamil Awad Shibli, Sergio Allegrini, Marcelo Yoshimoto, Bruno König

**Affiliations:** 1 Anatomy Department, Biomedical Science Institute, Universidade de São Paulo, São Paulo, Brazil; 2 Biotecnos Research Center, Santa Maria, Rio Grande do Sul, Brazil; 3 Catholic University of Uruguay, Montevideo, Uruguay; 4 Department of Periodontology and Oral Implantology, Dental Research Division, University of Guarulhos, Guarulhos, SP, Brazil; 5 Implantology Department, São Leopoldo Mandic, Campinas, Brasil; 6 Orthopedy Department, Ernst Moritz Arndt University, Greifswald, Germany; Faculté de médecine de Nantes, FRANCE

## Abstract

The present study investigated the moment of peak NF-kB activation and its dissipation in the cortical bone in the femur of Wistar rat stimulated by surgical trauma. Sixty-five Wistar rats were divided into 13 groups (*n* = 5 per group): eight experimental groups (expG 1–8) divided based on the euthanasia time point (zero, 1 h, 2 h, 4 h, 6 h, 8 h, 12 h and 24 h) and five sham control groups (conG 1–5) killed at zero, 1 h, 2 h, 4 h and 6 h, respectively. A 1.8-mm-diameter defect was generated 0.5 mm from the femur proximal joint using a round bur to induce the surgical trauma. Overall, the activation peak of NF-κB in the cortical bone was 6 h (expG5 group) independent of the evaluated position; this peak was significantly different compared to those in the other groups (p < 0.05). The surgical trauma resulted in a spread of immune markings throughout the cortical bone with an accentuation in the knee region. The present study provides the first evidence that the NF-κB activation peak was established after 6 hours in the cortical bone of Wistar rats. The signs from a surgical trauma can span the entire cortical bone and are not limited to the damaged region.

## Introduction

Bone is a highly specialized supporting framework characterized by its rigidity, hardness, and power of regeneration and repair. It protects the vital organs, provides an environment for marrow (involved in both blood forming and fat storage), acts as a mineral reservoir for calcium homeostasis and a reservoir for growth factors and cytokines, and plays a role in acid–base balance [1].

All cells in the body constantly communicate with each other under conditions ranging from normal physiological conditions to surgical or accidental injury. An array of regulatory proteins produced and secreted by lymphocytes and other cells play a role in the immune response cascade to trauma [2]. Following trauma, patients are subjected to dynamic alterations in the haemodynamic, metabolic, and immune responses that are largely orchestrated by endogenous mediators referred to as cytokines [3]. Numerous studies have shown that stimulation of a variety of inflammatory mediators takes place immediately after trauma [4,5].

Morphological and functional characterization has indicated the probability that the oscillation of fluid flow (OFF) inside of the canaliculus network causes damage to the osteocyte plasmatic membranes, thereby initiating a series of intracellular events involving the adaptation of the cell and tissue to a new physiological condition [6–8]. Similarly, the response type “shear stress" is an adaptive response to fluid flow and pressure variations on the vascular wall typically linked to haemodynamic conditions [9] that has also been identified in osseous cortex [6].

The nuclear factor kappa beta (NF-κB) is considered to be a stress sensor, although this issue remains unexplained despite an extensive range of studies [7] related to its role as a key regulator of inflammation, immune responses, cell survival, and cell proliferation [10–13]. NF-κB is a family of transcription factors that regulate many aspects of normal cellular functions as well as innate and adaptive immunity in response to pathogens and autoimmune stimuli [14,15]. The family includes NF-κB1 (also known as p50 and its precursor p105), NF-κB2 (p52 and its precursor p100), RELA, RELB, and c-REL. Homo- and heterodimers of these proteins activate transcription of target genes, typically through canonical (p50/RELA) and noncanonical (p52/RELB) signalling. NF-κB signalling (which typically refers to canonical RELA-mediated transcription) regulates many aspects of cellular activity [16]. Osteoclast differentiation is supported by cells of the osteoblast lineage that express the membrane-bound receptor activator (RANK) of RANKL (NF-kB ligand) and the macrophage colony stimulating factor (M-CSF) [17]; this process is also regulated by a secreted decoy receptor of RANKL (osteoprotegerin or OPG), which functions as a paracrine inhibitor of osteoclast formation [18]. The balance between OPG and RANKL regulates bone resorption and formation, and one imbalance of the RANKL/OPG system has been implicated in the pathogenesis of various primary and secondary bone malignancies [19].

Recently, an immunohistochemistry methodology was developed to analyse demineralization of the osseous cortex that aimed to expose the peak activation of NF-κB. The methodology not only indicated that the transcriptional factor was activated throughout the process of osseous cortex propagation but also demonstrated an increase in positive immune reactivity to this transcriptional factor in the knee joints in all studied groups [20]. This result indicates a possible joint degeneration mechanism in the knees (i.e., arthritis or arthrosis) due to the fact that the transcriptional factor is directly related to the stimulation of pro-inflammatory cytokines and osteoclastogenesis [21–23].

The objective of the present study was to investigate the moment of the peak activation of nuclear factor NF-kB and its dissipation in the cortical bone in the femur of Wistar rats stimulated by surgically induced trauma.

## Materials and Methods

### Animal model

Sixty-five Wistar male rats weighing ~350 g were divided into 13 groups (*n =* 5 per group). The eight experimental groups (expG 1–8) were divided in accordance with the euthanasia time: zero, 1 h, 2 h, 4 h, 6 h, 8 h, 12 h, and 24 h; the five control groups sham (conG 1–5) or false-operated groups were killed at zero, 1 h, 2h, 4 h, and 6 h. The sham group was performed only in these 5 times, because in a previous study performed and published to evaluate the technique used was found in the other time there was no mark [20], decreasing the number of animals used.

This study had been approved by the Ethics Committee in Animal Experimentation of the São Paulo University CEPA-IPEN/SP (# 049/09).

### Animal surgery

Each animal received sedation, analgesic and muscle relaxing through intramuscular injection conducted under anaesthetics including (2-2-xylidine)-5,6-dyhidro-4H-1,3-thyazyn Chlorate (Rompum, Bayer, São Paulo, SP, Brazil) (5.0 mg/kg) and Acepromazine (Acepran®1%—Univet, São Paulo, SP, Brazil) (0.75 mg/kg). For overall anaesthesia, IM ketamine (Ketamina®, Agener, União Química Farmacêutica Nacional SA, São Paulo, SP, Brazil) (35 mg/kg) was used. During the procedure, the animals were maintained under deep anaesthesia for 60 to 90 minutes. The surgery procedure was performed in the proximal femur region. The trichotomy was performed after anti-sepsis using iodopovidona. A skin incision was made and an incision was introduced into the fascia in the proximal-distal sense and, after the location of the knee joint, a metric marked ruler with drill gauge was used to determine the drilling point, and that was performed using one drill with a 1.8-mm diameter. Perforations were performed without irrigation to promote greater trauma in this area; then, the muscle tissue and skin were closed using a nylon 4.0 suture. In the sham (conG 1–5) or false-operated groups was made the incision and suture, and only no damage to bone tissue was performed. The rats were euthanized via decapitation with a guillotine.

### Sample processing

The samples were immersed in a fixative solution of 10% buffered formalin (0.1 molar phosphate buffer, pH 7.4), at 4°C for 7 days [20].

Following a wash in running water for 12 hours at room temperature, the samples were dehydrated in an ethanol series (70%, 80%, 90%, 95% and 100%) for 72 hours at each step. The dehydration was conducted at a temperature of – 20°C. After dehydration, the samples were rinsed in two xylol baths at – 20°C for 24 hours each step. This procedure removed the wax, allowing easy resin penetration and inhibition (Technovit® 7200 VLC, Kulzer & Co, Wehrhein, Germany). The resin inhibition was performed by immersing the samples in ethanol/resin solutions with proportions of 70%–30%, 50%–50%, 30%–70%, and 100%–100% for 72 hours at each step at – 20°C. After the polymerization conducted using a conventional process, the samples were placed into polyethylene and resin (Techonovit ® 7200 VLC) clusters at – 20°C and covered completely. The material was subjected to light polymerization for a period of 9 h under white light and an additional 90 h under blue light [20].

### Cutting and polishing

After polymerization, the samples were cut using a microtome (Exakt cutting equipment, Exakt Apparatebeau, Norderstedt, Germany) in accordance with the protocol of Donath & Breuner [24], and then worn and polished in a metallographic polisher (Panambra®). Prior to this, the samples were fixed in glass lamina with cyanoacrylate (Superbonder®) and worn and polished using 1200-mesh sandpaper dishes. The difference between wear and polish are defined based on the pressure variation due to the sample lamina. The sample thickness varied from 10 to 15 μm. The cutting, wearing and polishing were performed under a constant rinse.

### Acrylic stripping

Acrylic stripping was performed using 2-methoxyethyl acetate as the solvent; this step is fundamental for the initiation of the immunohistochemistry reaction. After complete acrylic stripping and assembly, the laminas were hydrated using an ethanol series (100%, 100%, 100%, 95%, and 85%) for 5 minutes each step, then further washed in a 100% ethanol solution supplemented with 10% ammonium hydroxide. Endogenous peroxidase activity was blocked by the immersion of the lamina two times in a 95% methanol and 20% hydrogen peroxide solution (50/50) for 15 minutes each. Then, the sample were placed in a distilled water bath and rinsed for 10 minutes in running water [20].

### Immunohistochemistry/NF-κB

For this process we used the streptavidin-biotin technique. Cuts with a width of 10 to 15 m were performed on the glass laminas previously prepared with 20% 3-aminopropyltriethoxy-silane (Sigma Chemical, St. Louis, MO, USA). For antigenic recovery, the laminas were immersed in citric acid (pH 6.0) for 30 minutes at a temperature range between 95°C and 98°C. The laminas were incubated with a polyclonal anti-NF–κB antibody (rabbit anti-human NF-*kappa* B, Lab. Zimed, 1:75). Subsequently, the samples were incubated with a secondary antibody and tertiary complex (LSAB+ Peroxidase, Dako Corporation, Carpinteria, USA). The reaction was detected by incubation with a solution of 0.025% diaminobenzidine (DAB) and serum albumin (BSA) (3,3'-diaminobenzidina, Sigma chemical, St. Louis, MO, USA) for three minutes, followed by dehydration, dialysis and mounting with Permount ® (Fisher scientific, Fair Lawn, USA).

### Analysis of the results

**Fig 1:**
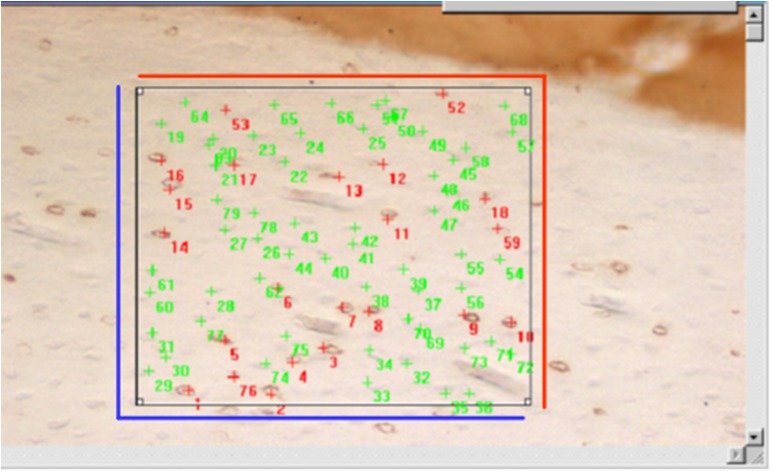
The image of previously standardized “Box” was included in the captured image to quantify the marked (in red) and unmarked (in green) cells.

The images obtained by microscopy (Eclipse E1000, Nikon, Japan) using a 10x objective were analysed using the image program *Image Pro Plus Version 4*.*1 for Windows*. Three different researchers manually performed the analysis for the positive and negative identifications of the presence of the transcriptional factor. A previously standardized “Box” was included in the captured image to quantify the marked and unmarked cells (Fig 1). Counting was repeated three times by three different researchers; therefore, the counting result corresponded to a triple blind test.

### Counting methodology

**Fig 2:**
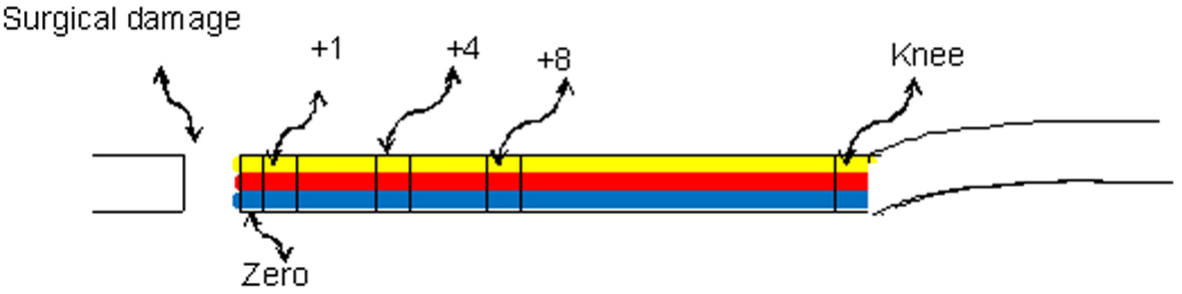
Schema indicating the counting positions (zero, +1, +4 +8 and knee) and the level of evaluations: periosteal (yellow), media (red) and endostea (blue).

The analysed sites were determined based on the distance from the location of the injury: the drilling site (position zero), 1 mm distant (position +1), 4 mm distant (position +4), 8 mm distant (position +8) and in the knee (position knee). In each of these positions, the samples were evaluated at three different levels: periosteal, media and endostea. The evaluation strategy is described in Table 1 and illustrated in the schema in Fig 2.

**Table 1:** The positions evaluated in each collected sample.

Region/Position	Pos 0	Pos +1	Pos +4	Pos +8	knee
Periosteal	Per/0	Per/+1	Per/+4	Per/+8	Per
Media	Med/0	Med/+1	Med/+4	Med/+8	Med
Endostea	End/0	End/+1	End/+4	End/+8	End

### Statistical analysis

1. Assessment to measure the relationship between NF-κb in position zero and knee using two correlation measurements:
  - A Pearson correlation considering -1 ≤ R ≤ 1 when R is near to ± 1 indicates that the studied variables are correlated. This indicates that there is a relationship among two variables; however, the relationship is linear.
  - A Spearman’s rank correlation measurement similar to the Pearson correlation measurement with (R) values among -1 ≤ R ≤ 1. This measurement is more comprehensive because in this study we assessed whether the relationship among the variables was non-linear and perhaps represented by a curve.
2. Assessment to analyse whether there are significant differences in the endostea, medium, and periosteal activation regions at all positions using a completely random model. The purpose of this model was to determine how the region behaves considering each of the studied factors (region and position distal P-D).

The ANOVA table was used to understand the model. If the interactions are significant, there should be an adequate region for comparison of each factor (region and P-D). If there is no statistical significance in the ANOVA table, the behaviour of the regions would be independent.

## Results

### Experimental group

The transcriptional factor was found throughout the osseous cortical, with a slight decline in the trauma regions and positions associated with the detachment trauma. There was an elevation of immune markings in the region proximal to the knee joint.

**Fig 3:**
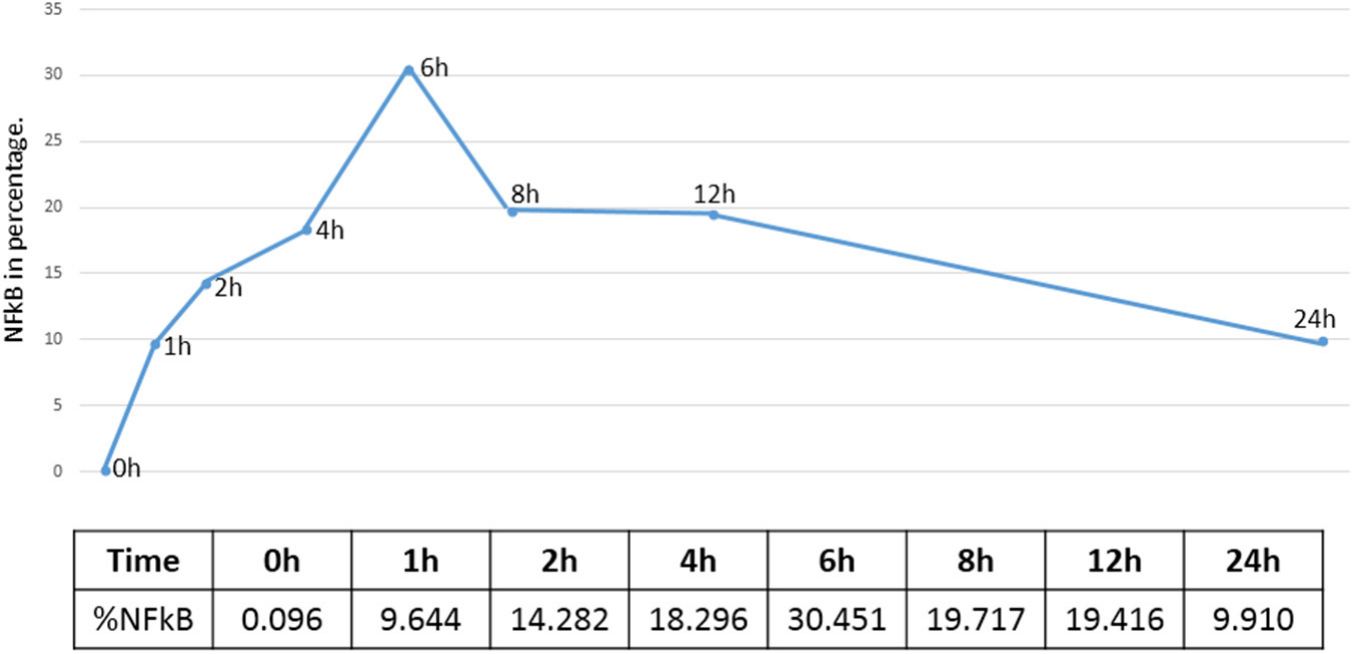
Graph of the average percentage of the experimental groups, indicating that the peak of NF-kB activation was observed in expG5 (6 h time point).

**Fig 4:**
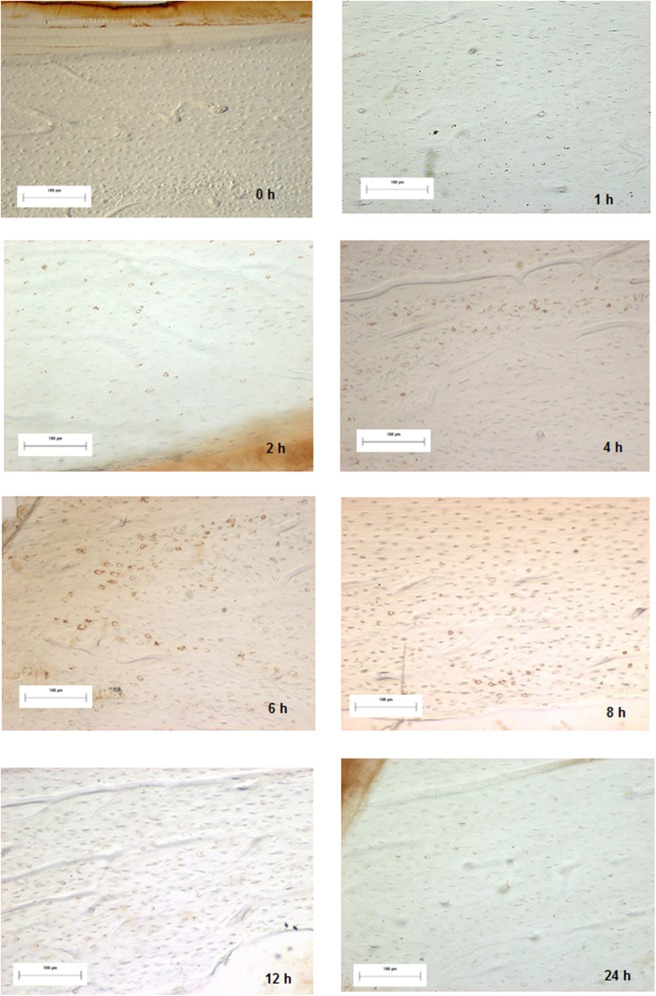
Images of all experimental groups in the periostal level of the different times in the position 0.

**Fig 5:**
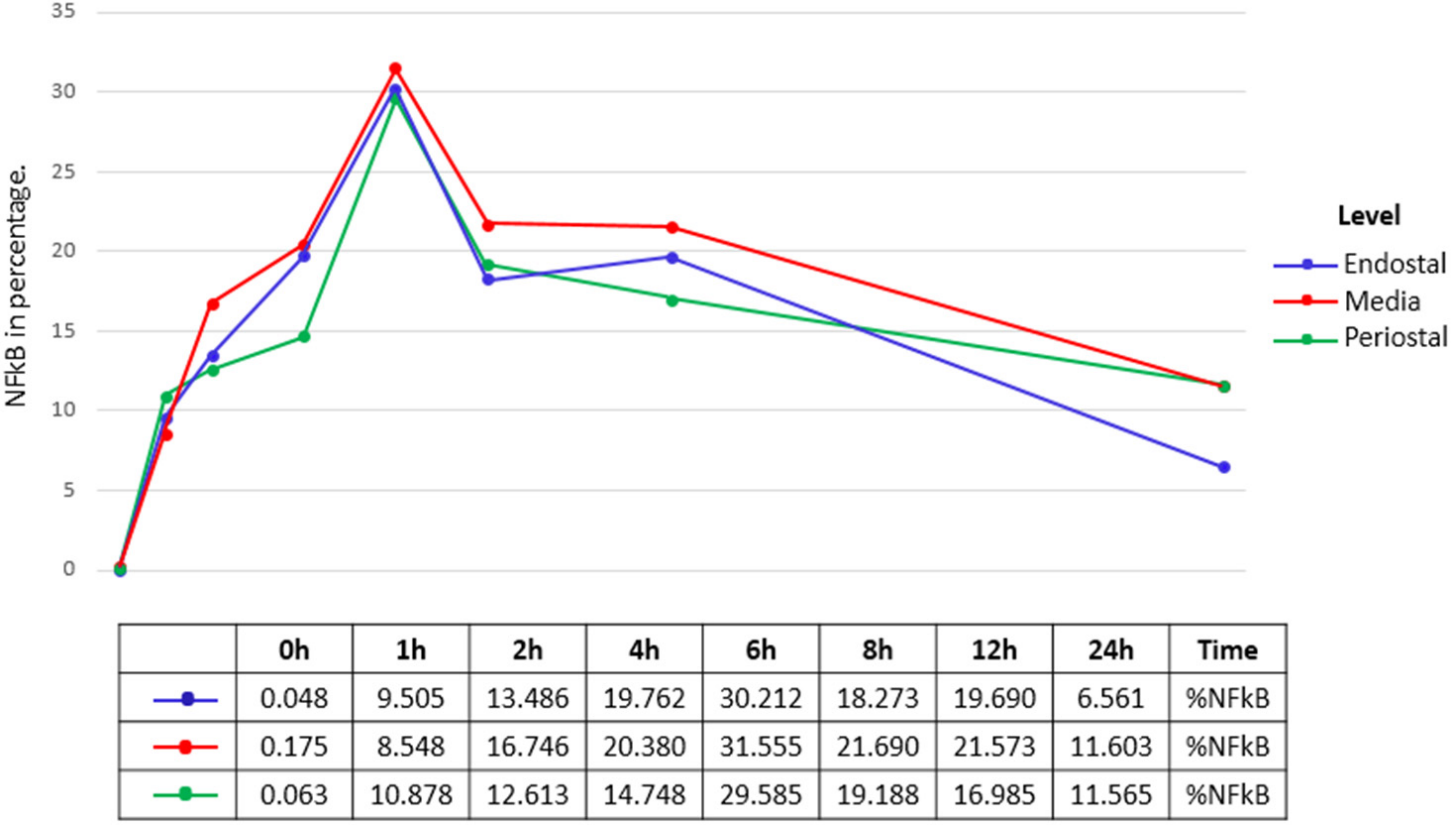
Graph of the behaviour at the three levels (endosteal, media and periosteal) of the proposed groups.

**Fig 6:**
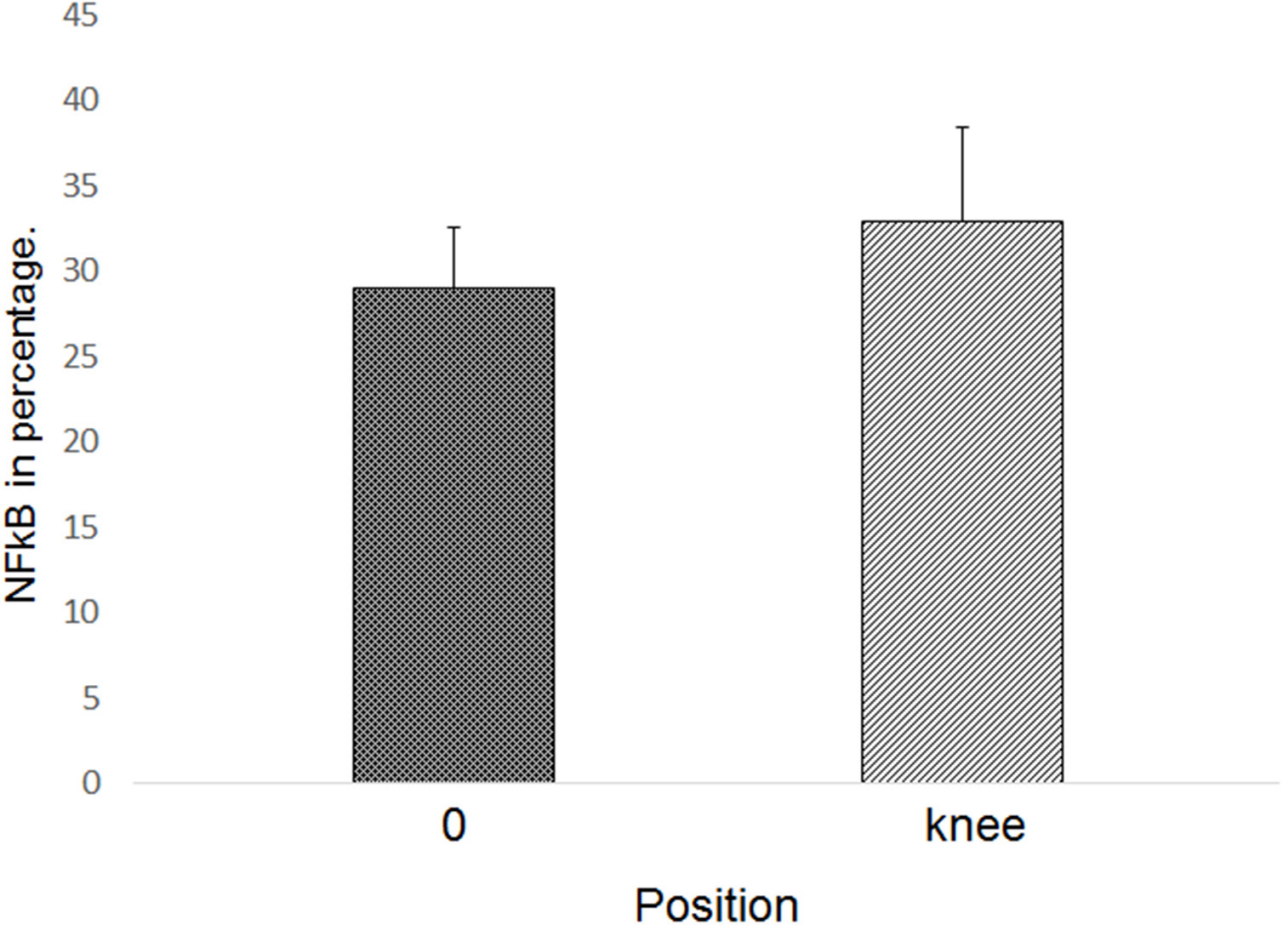
Bar graph showing the mean and standard deviation of the zero and knee positions in the expG5 group (6 hours).

Overall, the activation peak of NF-κB in the cortical bone was verified in the expG5 group (6 h) and was independent of the evaluated position, as shown in the graph in Fig 3 and the images of each group in the Fig 4. This peak was significantly different from the other groups (p < 0.05). The mean data at each position in each experimental group are demonstrated in Fig 5. There were no significant differences among the positions (p > 0.05). Following surgical trauma there was a spread of immune markings throughout the cortical bone, with an accentuation in the region of the knee (Fig 6). For this reason was selected for comparison positions 0 and knee.

**Fig 7:**
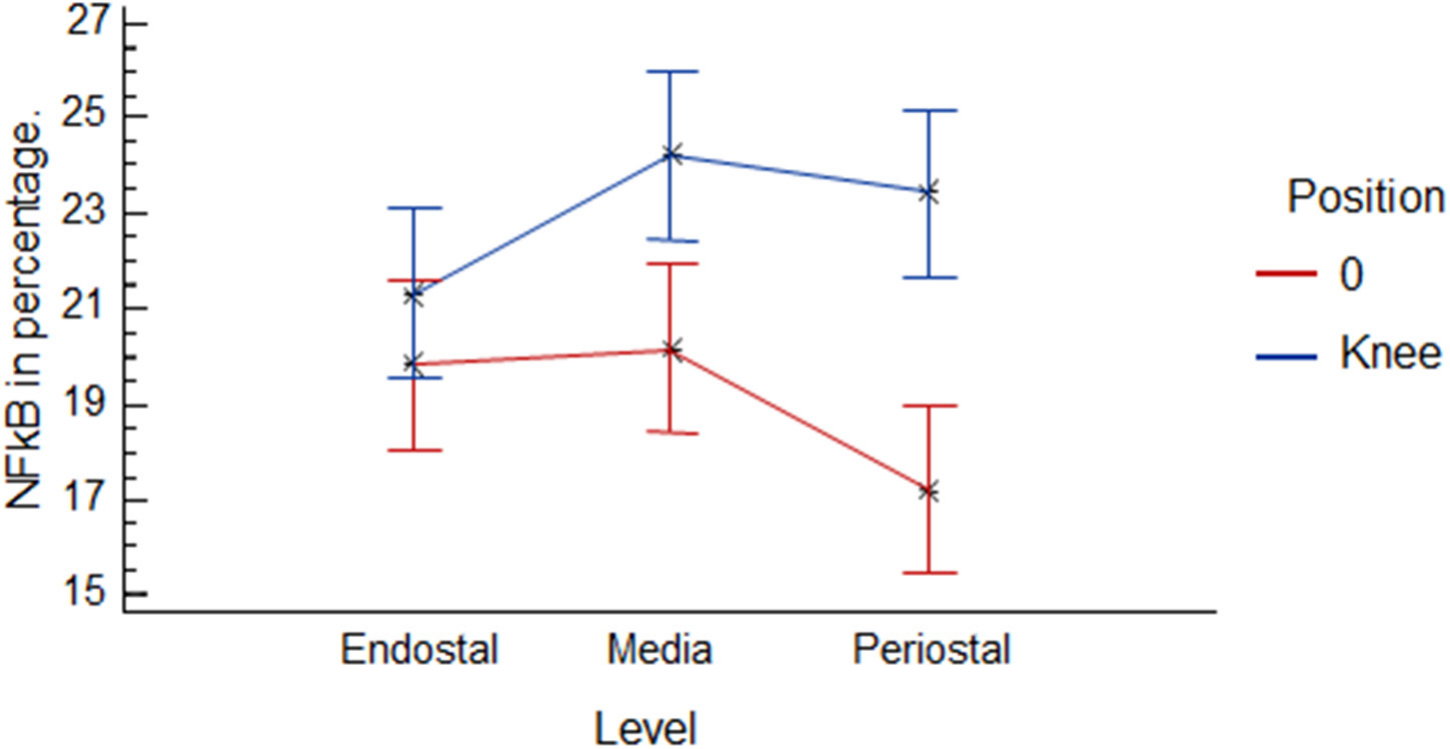
Average graph between the different levels observed at the zero and knee positions, indicating a more pronounced difference between the media and periosteal levels in the time of 12 hours.

**Fig 8:**
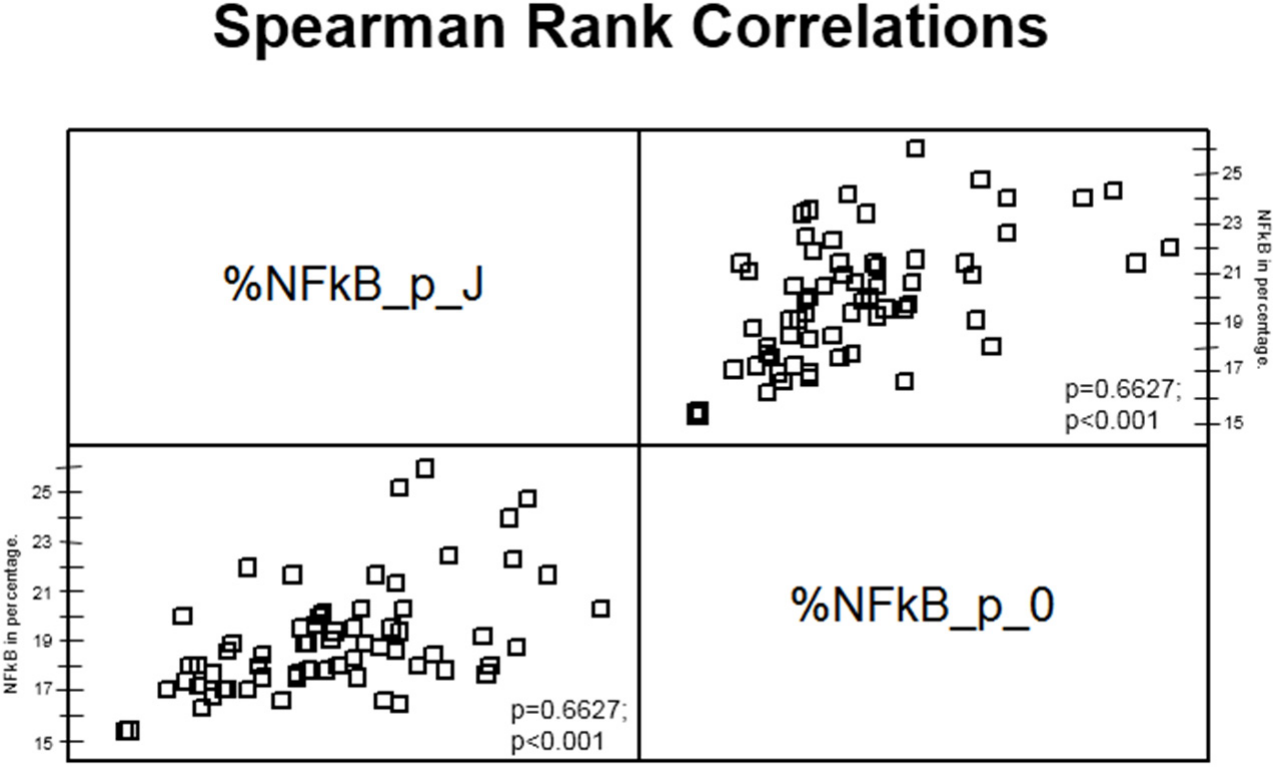
The Spearman rank correlations between each pair of variables. P-values below 0.05 indicate statistically significant non-zero correlations at the 95% confidence level.

**Fig 9:**
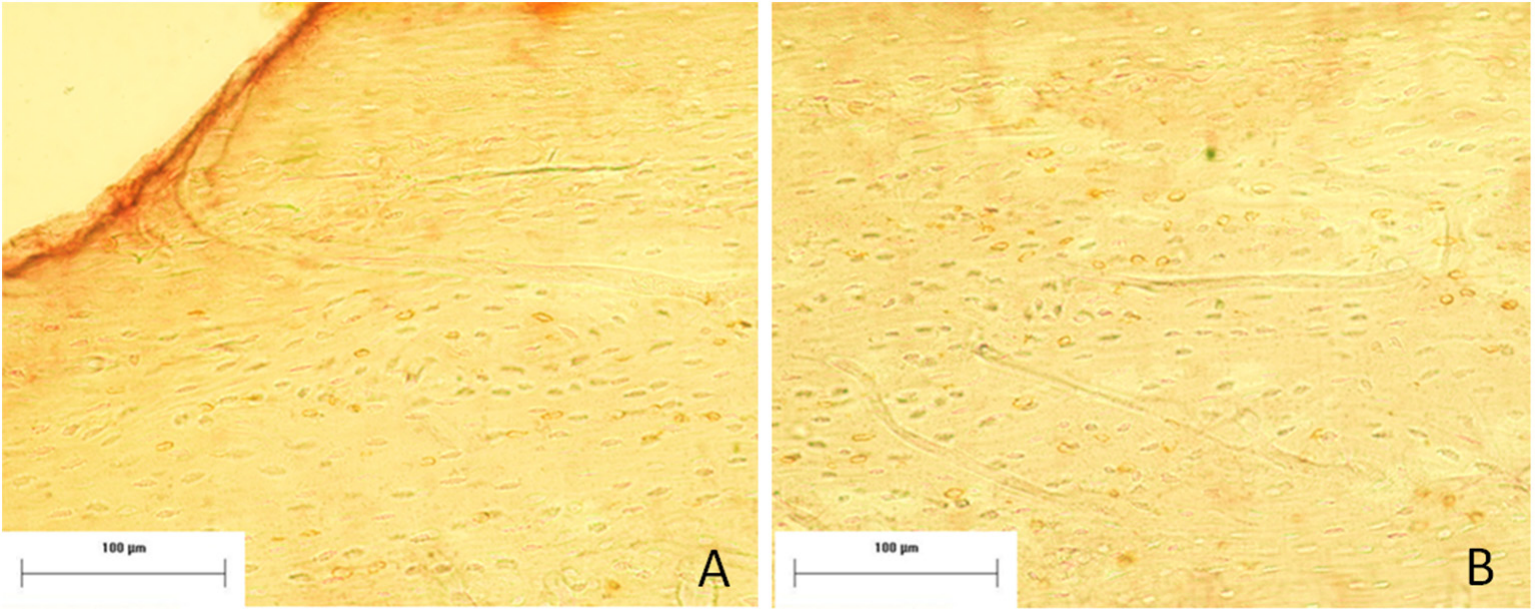
Image of the periosteal level in the expG5 group (6 hours) in the zero position (A) and knee position (B), demonstrating an increase in immunostaining for NF-kB in the knee position. Mayer´s hematoxylin staining.

Comparing the percent average at all time points, positions and regions (zero position versus knee position), there was a noticeable increase in immune markings in the knee region, but no statistical difference between these two positions (p = 0.2767). These differences were only statistically significant (p = 1.01^−5^) in the 12 h group (Fig 7). The linear correlation analysis among the zero and knee positions identified higher NF-κB activation in the zero position relative to the knee position. However, as noted, at all time points it was possible to verify that there were a higher number of immune markings in the knee position compared to position zero. The significant differences in anatomical positions are shown in Fig 8. This graphic shows Spearman rank correlations between each pair of variables, because in contrast to the more common Pearson correlations, the Spearman coefficients are computed from the ranks of the data values rather than from the values themselves. Consequently, they are less sensitive to outliers than the Pearson coefficients. However, this analysis indicated that there is a linear relationship among the two variables (p = 0.6461). In Fig 9A and 9B, it is possible to observe differences in immune markings in the same time period in zero and knee positions.

### Shan group

**Fig 10:**
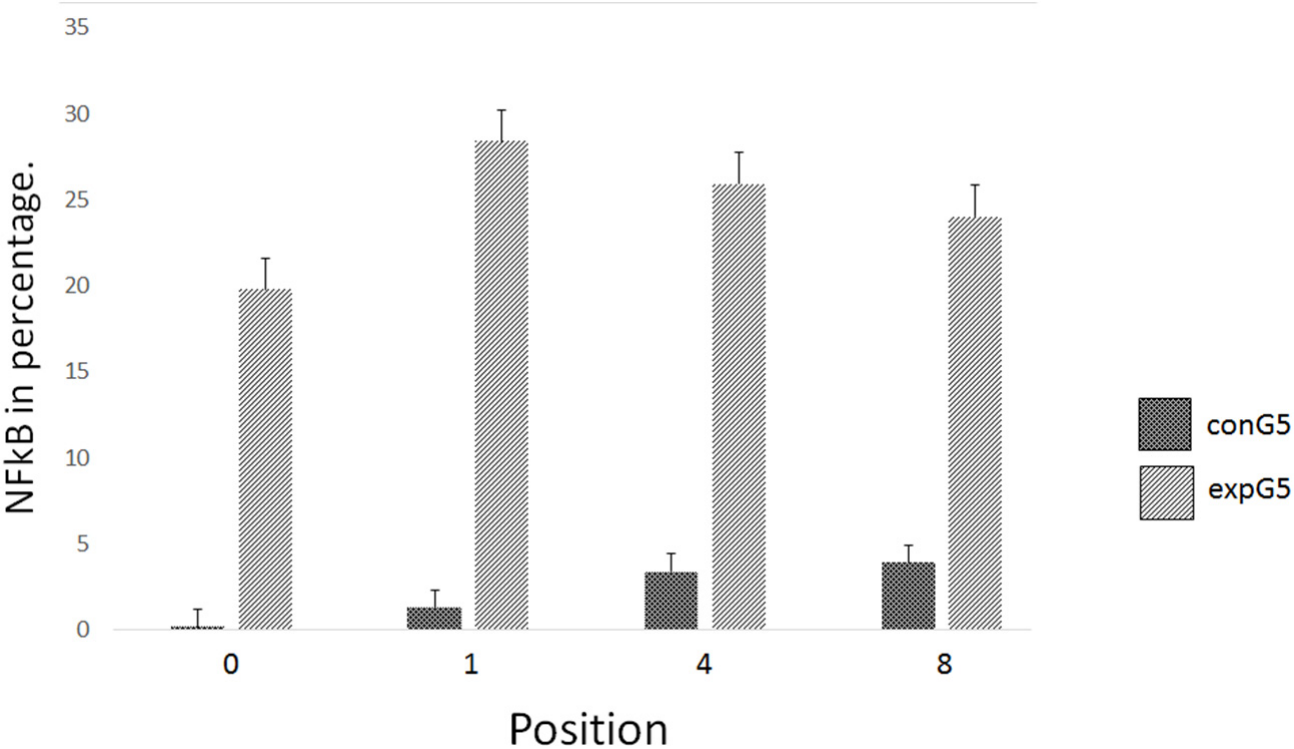
The graph bar indicate the mean and standard deviation of immunostaining at the periosteal level in the different positions studied comparing the conG5 and expG5 groups (6 hours).

**Fig 11:**
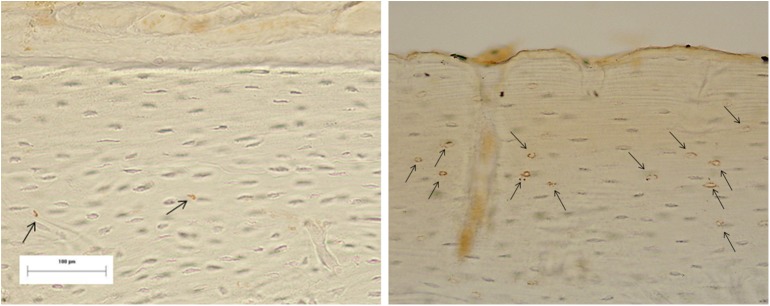
Image of immunostaining at the periosteal level (arrows) at 6 hours of the sham and experimental group, respectively.

**Fig 12:**
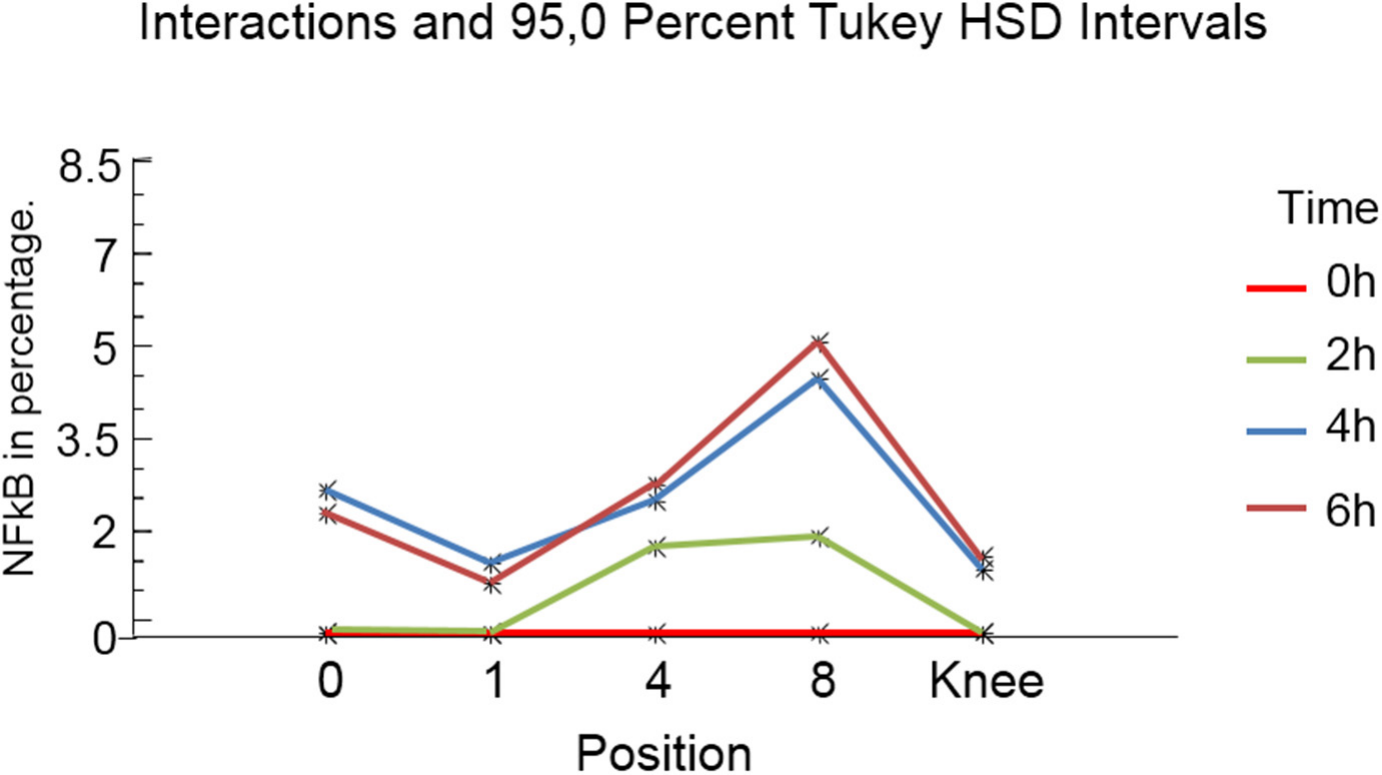
Graphical representation of the statistical results obtained in the periosteal level for the sham group in the different positions. It is possible to observe a greater concentration of immunostaining in positions 4 and 8, with a decline in the knee position.

Most of the immune markings remained in the periosteal region. In the media and endostea levels, the immune markings were scattered and in many cases did not fulfil the studied area and therefore were not quantified. This phenomenon was also observed for group zero in the experimental group. For this reason, we will present the statistical results only for the periosteal level (Fig 10). The results for this level display similarities comparative to the experimental group, although the results are clearly lower. In contrast to the experimental group, there was no correlation among the various studied levels and positions at the knee position. Moreover, the immune markings for NF-κB were restricted in the periosteal region and did not spread to other regions and positions (Fig 11). In Fig 12, it was possible to verify the statistical behaviour in different studied positions. There was a reduction in immune markings at the knee position and an increase in markings at positions 4 and 8. This behaviour could be related to the incision on the cortical bone performed during the surgical procedure.

## Discussion

The results demonstrate the spread of NF-κB throughout the osseous cortical and show that this spread is not limited to the region near the injury. Furthermore, this work shows an increase in transcriptional factor positive markings in the knee region. The data confirm the current knowledge level of osseous physiology. However, our study inserts them into the morpho-functional context and allows for the enrichment of a new scientific perspective.

Morphologically, osseous tissue (and in particular the osseous cortical) is constituted by lamellae circumscribed in a channel. The osteocytes in the lamellae communicate through their cytoplasmic extensions and constitute a real communication network that integrates all of the osseous cortical with the osteoblasts in the periosteal region through joint communicant [25].

The osseous cortical can be regarded as a transmission “information manager” from one region to another, and thus indicates the best tissue trauma reaction [26] as suggested by the results of this work. In the sham group, we observed a continuous location of NF-κB-positive markings near to the periosteal region without spread to other regions. The positive marking restriction opposes the proposed biophysical model [6,7]. This model indicates that the OFF inside of the canaliculus can change the membrane conformation because the signs are amplified for microfilament links among the cytoplasmic extensions present inside of the canaliculus and osseous matrix [27]. However, our results suggests that the amplification of this sign occurs through the communicant joints.

In the present experiment, we did not observe positive immune marking spread for NF-κB in the sham group at higher distances because the applied forces in this process are bio-physical (with respect to each trauma proportion). By definition, a given trauma is dependent on the physical forces that generate the sign dispersion process; these forces are sufficient for an effective sign dispersion and do not remain in the periosteal region as observed in our experiment.

The increasing statistical association of positive markings for NF-κB in the knee region showed a linear correlation; moreover, there was a significant difference between the zero and knee regions, with an increase in immune markings in the knee region in the Wistar rats in this study. Bradykinin released in the knee joint through afferent C fibres (component of the autonomous system) may activate the hypothalamic-pituitary-adrenal axis [28]. A previous evaluated the kinins released in the joints of Wistar rats after the animals were subjected to a stress situation (i.e., adrenalectomization and supplementation with anti-inflammatory steroids (glyco-corticoids)). The authors demonstrated that only the autonomic section component of bradykinin was effective in the knee joint inhibition of the animals submitted to the various treatments. This mechanism could explain in part the NF-κB activation in the knee joint because bradykinin has strong synergism with prostaglandin E2 and interleukin-1 resulting in the, release of the NF-κB receptor link (RANKL) [29].

Therefore, this neuroendocrine component could represent a serious candidate for understanding the physiological processes linked to the increase in immune markings found in the knee region. This is demonstrated in Fig 5 that presented a significant increase in the periosteal region in the knee position compared to the zero position.

To date, we have sought to understand the possible mechanisms that can lead to the transcriptional factor spread observed throughout the osseous cortex and its accumulation in the knee region. However, because the protein is strongly linked to inflammatory processes, osteoporosis (osteoclastogenesis) [21–23] and the immune system [30], we must consider some of the pathological aspects linked to our findings with degenerative processes, as discussed below.

The literature widely reports that NF-κB activation is related to the immune system and the production of pro-inflammatory cytokines, including IL-1, IL-6 and tumour necrosis factor-α (TNF-α), the chemokines IL-8, macrophage inflammatory protein-1α (MIP-1α) and molecules involved in adhesion (vascular cell adhesion-1 [VCAM-1] and intracellular cell adhesion molecule—1 [ICAM-1]), osteoclastic differentiation and activation of the RANK/RANKL/OPG system [30]. Therefore, neoformation and osseous resorption is mediated by these three factors [21,22]. In the same way, resorption modulated by RANK-RANKL in the joints is usually seen in osseous erosions arising from arthritis [31] and in part from post-menopause, when oestrogen does not inhibit IL-1. As a result, there is an increase in M-CSF that can lead to increases in bone resorption, thereby possibly promoting osteoporosis [32,33].

The expression of RANKL by the fibroblasts at the synovial membrane is stimulated by IL-1. In this way, it could act as a probable inductor mechanism for lesions in the knee joint via the osseous cortex. This finding has previously been verified in experimental work in Wistar rats, which demonstrated NF-κB spreading in the knee region [34]. This transcriptional factor can affect the expression of pro-inflammatory cytokines such as IL-1, which may induce synovial membrane fibroblasts to express the pro-inflammatory cytokine RANKL. RANKL signals macrophages to induce the differentiation of osteoclasts, resulting in cartilage erosion and/or osseous tissue, thereby contributing to joint degeneration [35,36].

This apparently simple work noted a series of events that have never before been described, and relied on comments from the pre-existing literature to formulate a hypothesis relating signalling to physiological propagation behaviour by the osseous cortex their accumulate in the knee joint region observed in this work to the pathological processes. The results indicating possible future studies to obtain a better understanding and possible treatments for inflammatory degenerative diseases related to bone tissue.

This study demonstrated only a part of the complex osseous mechanism. Based on our results, we can conclude that the NF-κB activation peak was established within 6 hours in femurs from Wistar rats. The signs from surgical trauma can span the entire osseous cortex, but are not limited to the damaged region. These NF-κB-positive immune markings for were increased in the knee region, indicating that osseous tissue works like a syncytium against external stimulus and may serve as a possible morphological indication of joint diseases related to the studied endonuclease.

## Conclusions

Within the limitations of this study, we could conclude that the NF-κB activation peak was established after 6 hours in the cortical bone of Wistar rats. The signs from surgical trauma can span the entire cortical bone and are not limited to the damaged region. The NF-κB-positive immune markings were increased in the knee region, indicating that bone tissue works like a syncytium against external stimulus.
